# Heating or ginger extract reduces the content of *Pinellia ternata* lectin in the raphides of Pinellia tuber

**DOI:** 10.1007/s11418-023-01717-7

**Published:** 2023-06-13

**Authors:** Yan Liu, Itsuki Nose, Kazuyoshi Terasaka, Tsukasa Fueki, Toshiaki Makino

**Affiliations:** 1 Department of Pharmacognosy, Graduate School of Pharmaceutical Sciences, Nagoya City University, 3-1 Tanabe-dori, Mizuho-ku, Nagoya, 467-8603 Japan; 2 Matsuya Pharmacy, 2927-5 Maki-kou, Nishikan-ku, Niigata, 953-0041 Japan; 3 Department of Traditional Medicine, Toho University School of Medicine, 5-21-16, Omori Nishi, Ota-ku, Tokyo, 143-8540 Japan

**Keywords:** *Pinellia ternata* lectin, Petroleum ether extraction (PEX), Pinellia tuber, Immunostaining, Processing, Acridity

## Abstract

Pinellia tuber, the dried tuber of *Pinellia ternata*, causes a very strong acridity sensation in the oral and laryngopharynx mucosa when taken orally in its unprocessed form. In traditional Chinese medicine (TCM), this sensation has been called "toxicity", and Pinellia tuber must be processed using ginger extract, licorice, or alum. In Japanese traditional Kampo medicine, since “toxicity” can be eliminated by decocting, it should not be processed. However, little is known about the mechanism underlying the "detoxification" of Pinellia tubers. In this study, we produced murine antiserum using recombinant *P. ternata* lectin (PTL), developed an immuno-fluorescence staining method for PTL in the needle-shaped crystals (raphides) that were prepared by petroleum ether extraction (PEX) from Pinellia tuber, and elucidated the mechanism of the processing of Pinellia tuber using heat or ginger extract. After heating the raphides in water, the amount of PTL contained in the raphides was significantly reduced by the immunostaining, although the shape of the raphides was not changed. Incubating raphides with dried ginger extract also significantly reduced the amount of PTL in the raphides in a concentration-dependent manner. By the activity-guided fractionation of ginger extract, the active ingredients in the ginger extract were oxalic acid, tartaric acid, malic acid, and citric acid. Among these four organic acids, oxalic acid mainly contributed to the effect of dried ginger extract by its content in ginger extract and its activity. These results exhibit scientific evidences for the traditional theories of processing to "detoxify" Pinellia tuber in TCM and Kampo medicine.

**Graphical abstract:**

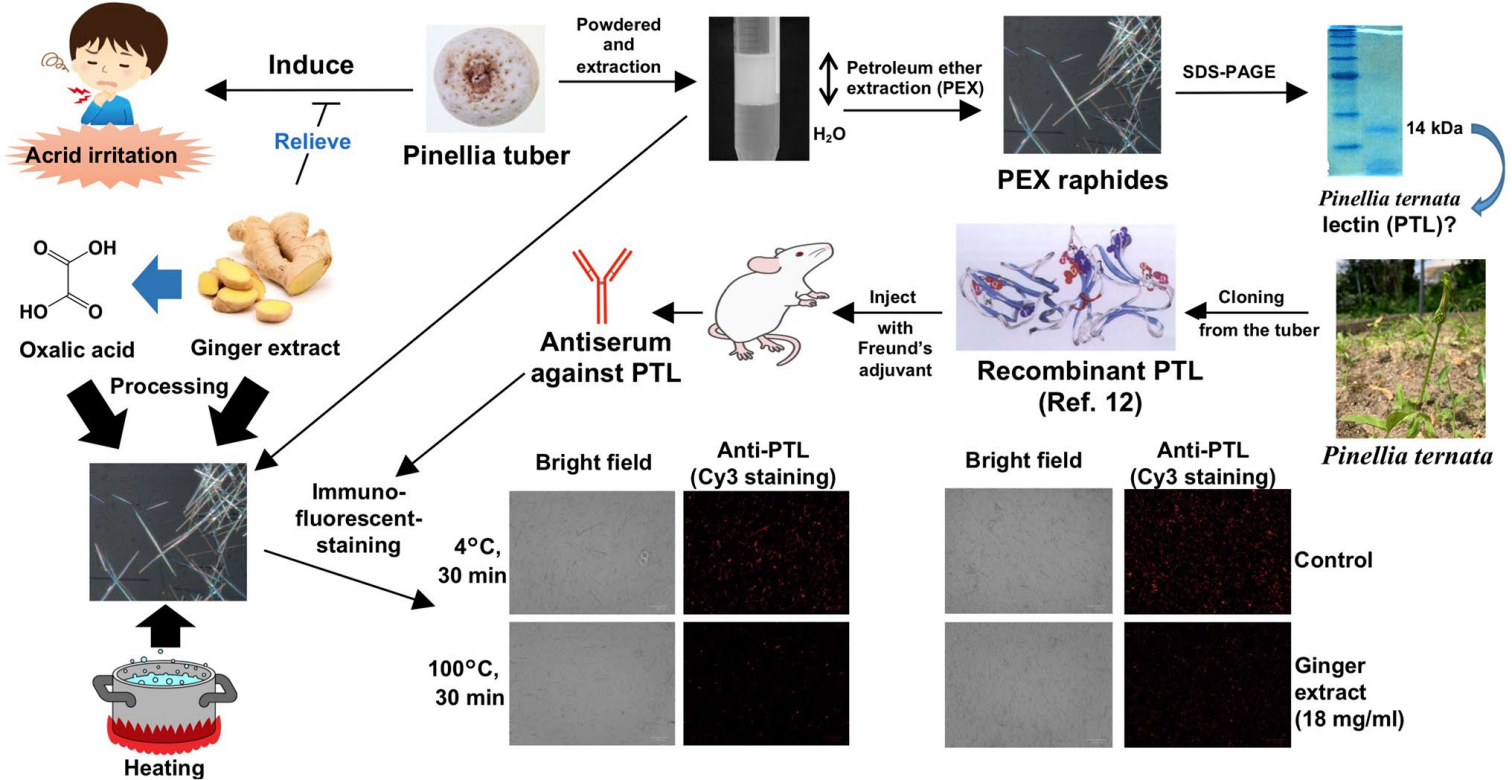

**Supplementary Information:**

The online version contains supplementary material available at 10.1007/s11418-023-01717-7.

## Introduction

Pinellia tuber, the dried tuber of *Pinellia ternata* (Thunb.) Makino (Araceae) belonging to the Araceae family, is registered as a crude drug in Japanese Pharmacopoeia 18th Edition [1] and Chinese Pharmacopoeia 2020 Edition [2]. Pinellia tuber is commonly prescribed in various formula such as hangeshashinto (banxiaxiexintang), hangekobokuto (banxiahoupotang) in both traditional Japanese Kampo medicine [3] and traditional Chinese medicine (TCM) [4]. Pinellia tuber has been used as anti-emesis, analgesic (angina, etc.), antitussive, regulating *fluid*, resolving *phlegm*, expectorant, resolving *fluid* retention in the stomach, descending *qi*, and drying dampness [5].

It is well known that Pinellia tuber causes a very strong acridity sensation at the oral and laryngopharynx mucosa when taken orally in its unprocessed form. According to the theory of TCM, this sensation has been called "toxicity", and Chinese Pharmacopoeia 2020 Edition describes that Pinellia tuber should be used after processing due to its toxicity, and registers three types of processed Pinellia tuber: "Fabanxia" (soaked in licorice decoction and lime water, then dried), "Jiangbanxia" (boiled with ginger and alum, then dried), and "Qingbanxia" (soaked in alum water or boiled with alum, then dried) [2]. According to TCM theory, the acridity of raw Pinellia tuber can be reduced by processing using ginger, licorice, alum, or lime. However, according to the theory of Japanese traditional Kampo medicine, the "toxicity" of Pinellia tuber can be eliminated by decocting and Pinellia tuber should not be processed to avoid weakening its effectiveness [6]. Pinellia tuber contains needle-shaped crystals made of calcium oxalate, and the acridity may be caused by these needles puncturing the cell membrane of the throat epithelial cells [7]. After processing Pinellia tuber, the structure of crystals changes and their content decreases, leading to a significant reduction in their acridity [8]. On the other hand, recent studies have shown that acridity is caused by insoluble needle-like raphides, which are composed of calcium oxalate and proteins [9].

In our previous study, we found that the surface of the raphides of Pinellia tuber had the characteristics of hydrophobicity, and reported that the raphides could be isolated from the petroleum ether (PE) layer in the partition of the suspension of the powder of Pinellia tuber against H_2_O [10]. In the present study, we estimated that the protein in the raphides of Pinellia tuber could be *P. ternata* lectin (PTL), and succeeded in the positive immunostaining of PTL on the surface of the raphides by developing murine antiserum against recombinant PTL protein. Furthermore, we elucidated the mechanism by which heating or processing with dried ginger extract reduced the content of PTL in the raphides.

## Materials and methods

### Materials

All crude drugs adhered to the quality control of the 18th Edition of the Japanese Pharmacopoeia [1]. The dried sliced form (approximately 2 mm wide) of Pinellia tuber (lot number, #009120002) and cut dried ginger (lot number, 8L25M) were purchased from Tochimoto Tenkaido (Osaka, Japan) and Daiko Shoyaku (Nagoya, Japan), respectively. Oxalic acid and L-( +)-tartaric acid were purchased from Fujifilm Wako Pure Chemical Corporation (Osaka, Japan). L-(–)-malic acid and citric acid monohydrate were purchased from Sigma Aldrich (St. Louis, MO, USA) and Nacalai Tesque (Kyoto, Japan), respectively.

## Preparation of petroleum ether extraction (PEX) raphides

PEX raphides were prepared according to the method described in our previous study [10]. The dried sliced form of Pinellia tuber (90 g) was powdered using an electric mill, suspended in H_2_O (250 ml), and stood at room temperature for 60 min. Then, PE (60 ml) was added and mixed vigorously. The suspension was kept at 4 °C for 90 min, and three layers appeared. When shaken gently, the raphides in the middle layer moved into the upper PE layer. The cloudy upper layer was immediately collected to avoid contamination of the remaining middle layer. This collection procedure was repeated twice with fresh PE (50 ml), and all PE layers were combined and centrifuged at 1.0 × 10^3^ × *g* for 5 min. The supernatant was removed, and the precipitated raphides were washed three times with fresh PE (40 ml), and then three times with H_2_O (20 ml). The raphides were suspended in PE (15 ml) to create the PE extraction (PEX) raphide suspension, and stored at − 20 °C. A part of the suspension was lyophilized, and the dried weight was measured. The concentration of the PEX raphide was 26 mg/ml. Another part of the suspension was homogenized in phosphate-buffered saline (0.15 M, pH 7.2, PBS), and the concentration of protein was estimated at 1.9 mg/ml using the BCA protein assay kit (Thermo Fisher Scientific, Waltham, MA, USA). The procedure is summarized in Supplementary Figure S1.

## Sodium dodecyl sulfate (SDS)-polyacrylamide gel electrophoresis (PAGE) of PEX raphides protein

PEX raphides for SDS-PAGE were prepared as described in the previous section. The powdered Pinellia tuber (80 g) was suspended in H_2_O (240 ml) and PE (80 ml), and mixed vigorously. The raphides were collected and washed with fresh PE (20 ml) three times, and then with H_2_O (10 ml) three times. The raphides were finally suspended in PE (10 ml) and transferred into a beaker, and PE was utterly evaporated using a stream of air. The aqueous solution of 0.1 M AlCl_3_ – 0.15 M KAl(SO_4_)_2_ (20 ml) was added and stirred very vigorously for 12 h until the raphides disappeared. The solution was then extracted with PE (20 ml) three times. All PE layers were merged and evaporated to obtain a white solid residue. The residue was dissolved in 0.5 ml of 5% SDS solution, and the concentration of protein was measured using a BCA protein assay kit. The protein (25 µg) was applied for SDS-PAGE analysis using 15% acrylamide gel. Gel was stained with coomassie brilliant blue, and a band at 14 kDa appeared. The band was cut and ordered for peptide sequence analysis (Japan Institute of Leather Research, Tokyo, Japan).

## cDNA cloning and recombinant protein expression of PTL

The whole plant of *P. ternata* was harvested from Nagoya City University Medicinal Plant Garden in August 2020. Total RNA was extracted from the tuber using innuPREP Plant RNA Kit (Westburg Life Sciences, Utrecht, The Netherlands), and the first-strand cDNAs were synthesized from total RNA using SuperScript IV Reverse Transcriptase (Thermo Fischer Scientific). The primers for open reading frame sequence were designed based on the PTL mRNA complete coding sequence (GenBank, EU199445.1), and after successful amplification of the target sequence, the purified PCR product was subcloned into the pMD20 T-vector (Takara Bio, Kusatsu, Japan) and the DNA sequences were analyzed.

The sequence for the putative mature polypeptide of PTL was amplified and subcloned into pET45b (Merck, Darmstadt, Germany) and expressed in *E. coli* BL21(DE3) (Thermo Fischer Scientific). The expression and purification of recombinant proteins were performed as previously described [11]. Primers used in cDNA cloning and vector construction are indicated in Supplementary Table S1.

Affinity-purified recombinant PTL protein solution was diluted with Milli-Q water to a final volume of 10 ml, and dialyzed (cut off 12–14 kD, Spectra/Por 4 dialysis membrane, Spectrum Laboratories, Rancho Dominguez, CA, USA). The resulting product was lyophilized, weighed, and reconstituted in PBS to make a solution (1.0 mg/ml).

## Preparation of antiserum using recombinant PTL

Ten female Balb/c mice (5 weeks old) were obtained from Japan SLC (Shizuoka, Japan). Recombinant PTL protein was suspended in Freud's complete adjuvant (Sigma), and intraperitoneally (*i.p.*) injected into mice (25 µg of PTL per body), and *i.p.* injected again 2 weeks after the first immunization. Four and 6 weeks after the first immunization, recombinant PTL protein suspended in Freud's incomplete adjuvant (Sigma) was *i.p.* injected into mice (12.5 µg of PTL per body). Seven weeks after the first immunization, mice were anesthetized with pentobarbital (40 mg/kg, *i.p.*) and blood was collected from the heart. The serum was obtained by centrifugation, merged, and stored at – 70 °C. The experimental procedures were conducted in accordance with the Nagoya City University Guidelines for the Care and Use of Laboratory Animals, and the study protocol (R4-P-02) was approved by the local Animal Ethics Committee of Nagoya City University.

Dot blot analysis was performed using this antiserum. PEX raphides homogenate in PBS (dried weight/volume, 0.26 mg/ml, 26 and 2.6 µg/ml) and the supernatant (5 µl each) described in the next section were spotted onto Immobilon-E® membrane (Merck). After blocking the membrane using Blocking One Reagent® (Nacalai), the membrane was incubated with this antiserum (1:1000) diluted with 10% Blocking One Reagent® in Tris-buffered saline with 0.1% Tween 20 (TBS-T) at 4 °C overnight. After washing the membrane with TBS-T for three times, the membrane was incubated with horseradish peroxidase (HRP)-labeled anti-mouse IgG antibody (Cell Signaling Technology, Danvers, MA, USA) (1:1000) diluted with 10% Blocking One Reagent® in TBS-T at 4 °C for 1 h. After washing the membrane with TBS-T for 3 times, the membrane was colored with Immobilon® Crescendo Western HRP substrate (Merck) and the chemiluminescence was detected using Amersham Imager 600 (GE HealthCare, Chicago, IL, USA).

## Immunostaining of PTL in PEX raphides

PEX raphide suspension (0.13 mg, 5 µl) was diluted with PBS (95 µl), and the sample solution in PBS or PBS containing 1% DMSO (100 µl) was mixed and incubated at 4, 40, or 100 °C for 30 min. The suspension was then centrifuged at 3.0 × 10^3^ × *g* for 10 min, and the supernatant was removed. The raphides were incubated with Blocking One Reagent® at room temperature for 1 h. After centrifugation, the supernatant was collected and used for dot-blot analysis. The raphides in the residue were incubated with antiserum against PTL diluted with 10% Blocking One Reagent® in TBS-T (1:40) at room temperature overnight. After centrifugation and removal of the supernatant, the raphides were incubated with TBS-T for 10 min and this step was repeated three times. The raphides were incubated with anti-mouse IgG-Cy3 antibody (Proteintech, Rosemont, IL, USA) diluted with 10% Blocking One Reagent® in TBS-T (1:100) at room temperature for 1 h. After centrifugation and removal of the supernatant, the raphides were incubated with TBS-T for 10 min, and this step was repeated three times. The raphides were suspended in PBS (50 µl), and 15 µl of the suspension was dropped onto a slide glass and covered with a cover glass. The microscope image was monitored using a ZOE Fluorescent Cell Imager (BioRad, Hercules, CA, USA), and the photos of three different fields were taken by normal light and by the red fluorescence channel. The area of raphides in the bright field photo and that of the red part in the fluorescence photo were measured using Image J (https://imagej.nih.gov/ij/download.html) and the ratio of the red part to the area of raphides was calculated. The average of the ratio among 3 different fields was used as the data of individual raphides sample. Their half-maximum inhibitory concentration (IC_50_) value was calculated by the least square regression line made from 3 points that crossed at 50% of the percent of the inhibition value and the logarithmic concentration values.

## Preparation and partition of dried ginger extract

Cut-dried ginger (16 g) was mixed with H_2_O (160 ml) and heated at 100 °C for 30 min. After centrifugation at 1.0 × 10^3^ × *g* for 10 min, the supernatant was lyophilized to yield a dried powdered extract (2 g). The extract was dissolved in ion-exchanged water at a concentration of 400 mg/ml, and stored at − 20 °C. For immunostaining, dried ginger extract was reconstructed in PBS.

Dried ginger extract aqueous solution (36 mg/ml, 300 µl) was mixed with ethyl acetate (200 µl) and vigorously vortexed. After centrifugation at 1.2 × 10^3^ × *g* for 5 min, the ethyl acetate layer was collected, and the water layer was mixed with ethyl acetate (200 µl) and vortexed again. This was repeated twice, and the ethyl acetate layers were merged. The remaining water layer was mixed with 900 µl of ethanol, vortexed well, and incubated at – 20 °C for 30 min. After centrifugation at 1.2 × 10^3^ × *g* for 5 min, the supernatant was collected. Ethanol (300 µl) was added into the residue, and centrifugated at 1.4 × 10^4^ × *g* for 5 min, and this step was repeated twice, and the supernatants were merged. Dried ginger extract aqueous solution (36 mg/ml, 300 µl), ethyl acetate layer, ethanol-soluble part, and ethanol-insoluble part were incubated at room temperature overnight under N_2_ gas flow to evaporate organic solvents, and then lyophilized. The residues were resuspended in 300 µl of PBS containing 1% DMSO. The fractions of dried ginger extract as 36 mg of original ginger extract/ml (100 µl) were mixed with PEX raphide suspension (5 µl) and PBS (95 µl), incubated at 40 °C for 30 min and immuno-stained as described above.

## Determination of organic acid content in dried ginger extract

The dried ginger extract and organic acids were dissolved in H_2_O. After centrifugation at 1.4 × 10^4^ × *g* for 7 min, the supernatants were applied into the HPLC system (Shimadzu LC–10A_*VP*_, Kyoto, Japan) under the following conditions. Condition 1; column, Cosmosil 5C_18_-PAQ (150 mm × 4.6 mm i.d., Nacalai); mobile phase: 10 mM phosphate buffer (pH 2.5), 1.0 ml/min; detection, UV 210 nm; column temperature, 30 °C; citric acid (4.3 min). Condition 2; column, Cosmosil HILIC (250 mm × 4.6 mm i.d., Nacalai); mobile phase, 10 mM phosphate buffer (pH 6.8)/acetonitrile (70:30), 1 ml/min; detection, UV 203 nm; column temperature, 40 °C; oxalic acid (8.7 min).

Since malic acid and tartaric acid were not well separated under the conditions described above, LC–MS/MS system (Quattro Premier XE (Waters Corporation, Milford, MA, USA) was used with the following conditions: column, Intrada Organic Acid (50 × 2 mm i.d., Imtakt, Kyoto, Japan); mobile phase (A) acetonitrile/H_2_O/formic acid = 10/90/0.1, (B) acetonitrile/100 mM ammonium formate = 10/90, 0% B (0–1 min), 0–100% B (1 – 7 min), 0.2 ml/min; Detection, electrospray ionization (ESI) (–)—single ion monitoring (SIM), malic acid (*m/z* = 133.0, 1.1 min) and tartaric acid (*m/z* = 149.0, 1.9 min); column temperature, 23 °C.

Quantitative analysis was performed using an external standard method with standard solutions containing each organic acid (citric acid, 500 ng, 1.0, and 2.0 μg; oxalic acid, 16, 80, 400 ng, and 2.0 μg; malic acid and tartaric acid, 10, 30, 60, and 100 ng, respectively). These standard compounds and dried ginger extract (40 µg for citric acid; 16 µg for oxalic acid; 1.83 µg for malic acid; 3.66 µg for tartaric acid) were applied into HPLC system, and linear regression of the concentration range of standard compounds by the peak-area was calibrated with the least-squares method (*r*^*2*^ = 0.999). The concentration of organic acids in the dried ginger extract was calculated by this calibration formula.

## Statistical analysis

The results were presented using a bar graph for the means and individual values were plotted. Student's *t*-test was used for the comparison between two groups. For multiple group comparisons, Dunnett's test was used for multiple comparisons against the control group, and Bonferroni’s multiple comparison test was used for multiple comparisons between all pairs. All analyses were conducted using Mac Statistical Analysis Ver 3.0 (Esumi, Tokyo, Japan). Data are expressed as mean ± standard deviation (S.D.).

## Results

### Identification of the protein from PEX raphides of Pinellia tuber

**Fig. 1:**
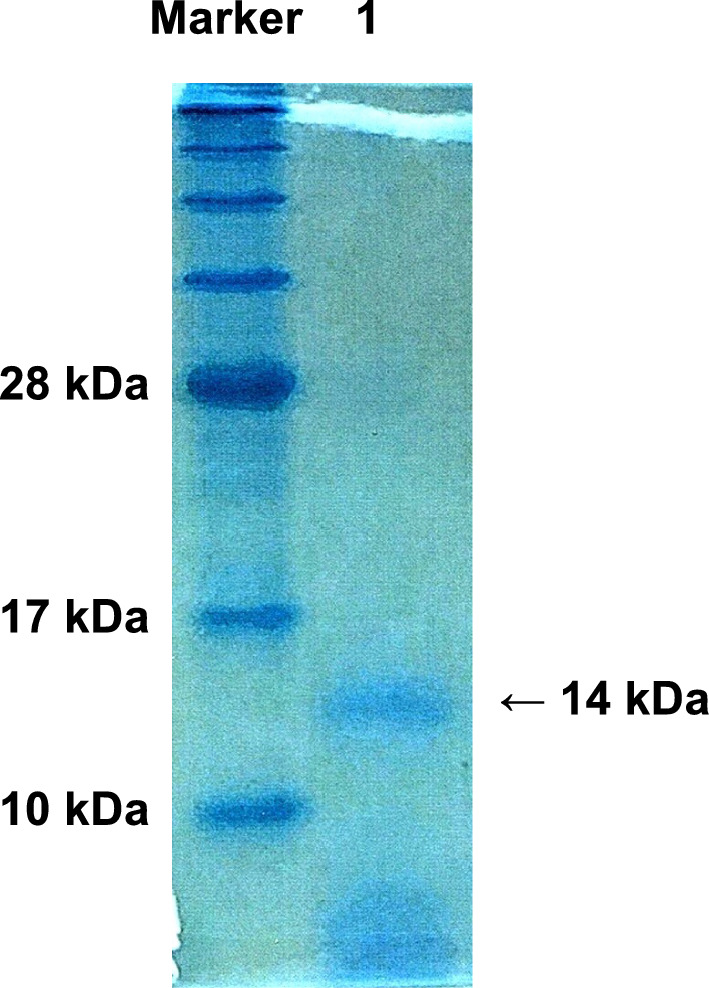
SDS-PAGE of the protein from PEX raphides of Pinellia tuber (PEX protein) using 15% acrylamide gel. PEX raphides were dissolved in 0.1 M AlCl_3_–0.15 M KAl(SO_4_)_2_ and the protein was extracted with PE. The residue was dissolved in 5% SDS, and applied for SDS-PAGE analysis (lane 1).PEX, petroleum ether extraction

The proteins prepared from PEX raphides were separated using SDS-PAGE and stained by coomassie brilliant blue. According to the molecular weight marker, a band at approximate 14 kDa was found in PEX proteins (Fig. 1).

**Fig. 2:**
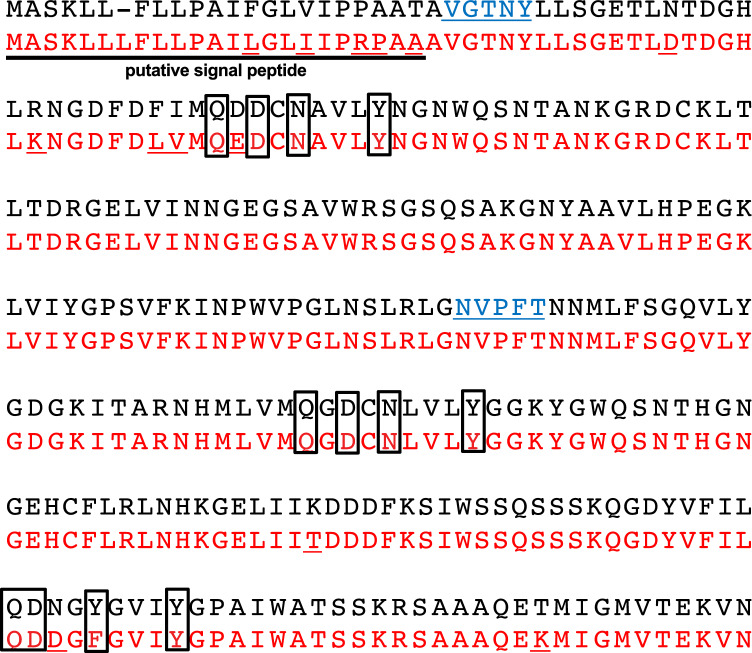
Comparison of amino acid sequences between EU199445.1 and *Pinellia ternata* lectin (PTL) isolated from the present study. The band in the gel at 14 kDa shown in Fig. 1 was cut and ordered for the peptide-sequencing. The sequences of EU199445.1 and PTL (LC764429) isolated in this study were shown in black and red, respectively. Amino acids shown in blue letters are detected by peptide sequences of the band shown in Fig. 1. The differences between two sequences are underlined. The boxes refer to mannose-binding sites analyzed using secondary and three-dimensional structures in the previous study [12]

To identify the molecules of this band, we ordered commercial peptide sequence analysis. The results indicated that this band contained two different proteins, and the amino acid sequences detected were #1, N and V; #2, V and G; #3 P and T; #4, F and N; #5, T and Y from N-terminal. We searched the combinations of these amino acid sequences and finally found that this band may contain the components of PTL [12] (Fig. 2).

## Cloning of PTL from the tubers of* P. ternata*

We isolated a full-length cDNA encoding PTL from the fresh tubers of *P. ternata* by reverse transcription (RT)-PCR using the primer pair designed from the nucleotide sequences of the PTL mRNA complete coding sequence. The isolated PTL cDNA was 810 bp, which encoded a polypeptide composed of 269 amino acids and the expected molecular weight was 29.3 kDa. In a comparison of this polypeptide with PTL in Genbank, agglutinin from *P. ternata* (QNL35375.1) shared 97% identity. This PTL had a mutation of 14 amino acids compared to the published peptide sequence [12] shown in Fig. 2. The nucleotide sequences of PTL are provided in the DDBJ DNA Database under the accession numbers shown in the legend for Fig. 2. To obtain the recombinant PTL, a truncated PTL without the putative signal peptide was subcloned into pET45b vector and expressed in *E.coli* BL21 (DE3). By SDS-PAGE analysis, we found a band at 28 kDa expected as PTL, and then recombinant PTL protein was successfully purified using the Ni–NTA affinity column.

## Preparation of antiserum using recombinant PTL in mice

Using recombinant PTL protein, we prepared antiserum against PTL using mice. By dot blot analysis, we confirmed that this antiserum bound to PEX raphides homogenate in concentration-dependent manners (Supplementary Fig. S2A).

## Immunostaining for PTL in PEX raphides with or without heating

**Fig. 3:**
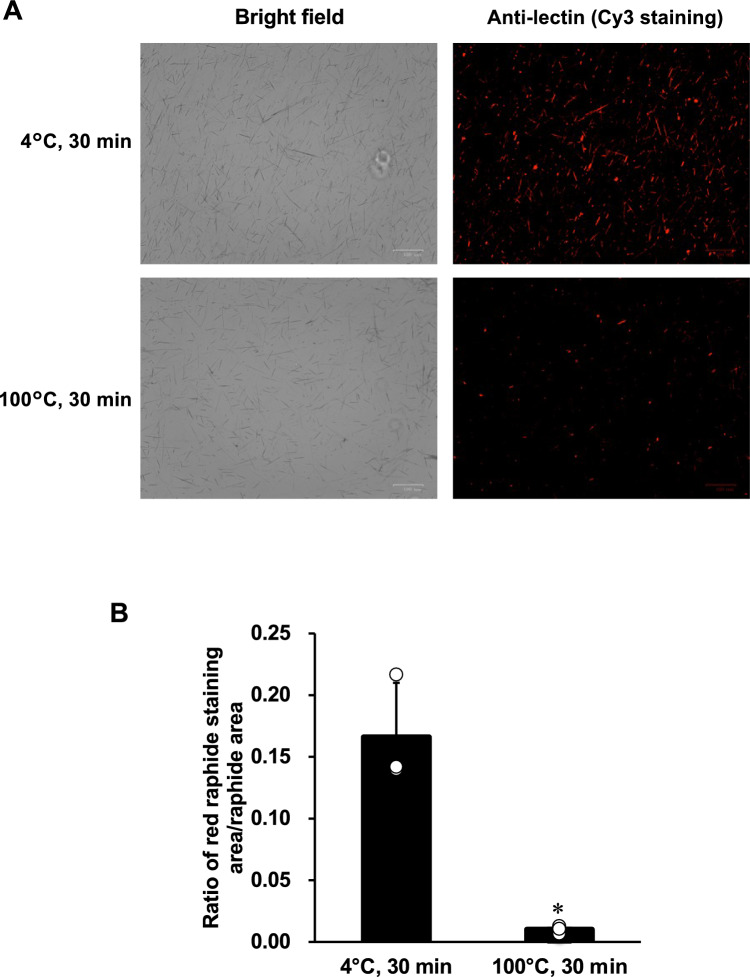
Immunostaining of PEX raphides of Pinellia tuber with or without heating using antiserum against *Pinellia ternata* lectin (PTL). PEX raphides were suspended in phosphate buffered saline (PBS) and incubated at 4 or 100 °C for 30 min. Then, PEX raphides were stained with murine antiserum against PTL and Cy3-conjugated secondary antibody. **A** Photo of light and fluorescence microscopies. **B** The black area (raphides) in the photo of the light microscopies and the red area (PTL) in that of fluorescence microscopies were measured in the same position using Image J. The ratio of the red staining area to the raphide area was calculated. The results were presented as a bar graph showing the mean and individual values plotted ± standard deviation (S.D.) (*n* = 3). *p < 0.05 compared to 4 °C group by Student's *t*-test. PEX, petroleum ether extraction

We conducted immunostaining of PEX raphides using this antiserum. As shown in Fig. 3A, PTL could be detected at the surface of PEX raphides by immuno-fluorescence staining. When the PEX raphides were heated at 100 °C for 30 min, the amount of fluorescence at the raphides decreased compared to that in the control PEX raphides treated at 4 °C for 30 min, although the shape and number of PEX raphides detected under bright light microscopy did not change (Fig. 3A). The immuno-dot blot analysis of the supernatants of the raphides suspension revealed that PTL was released from the raphides into the supernatant by heat treatment (Supplementary Fig. S2). The relative area of the staining in PEX raphides was analyzed using Image J. Based on the observation of the raphides area under bright light using the same parameters, the staining area on the raphides after being heated at 100 °C for 30 min was significantly lower than that of the control kept at 4 °C (Fig. 3B). In another experiment, the raphides treated without this antiserum with the secondary antibody did not show any fluorescence (data not shown).

## Immunostaining for PTL in PEX raphides treated with dried ginger extract

**Fig. 4:**
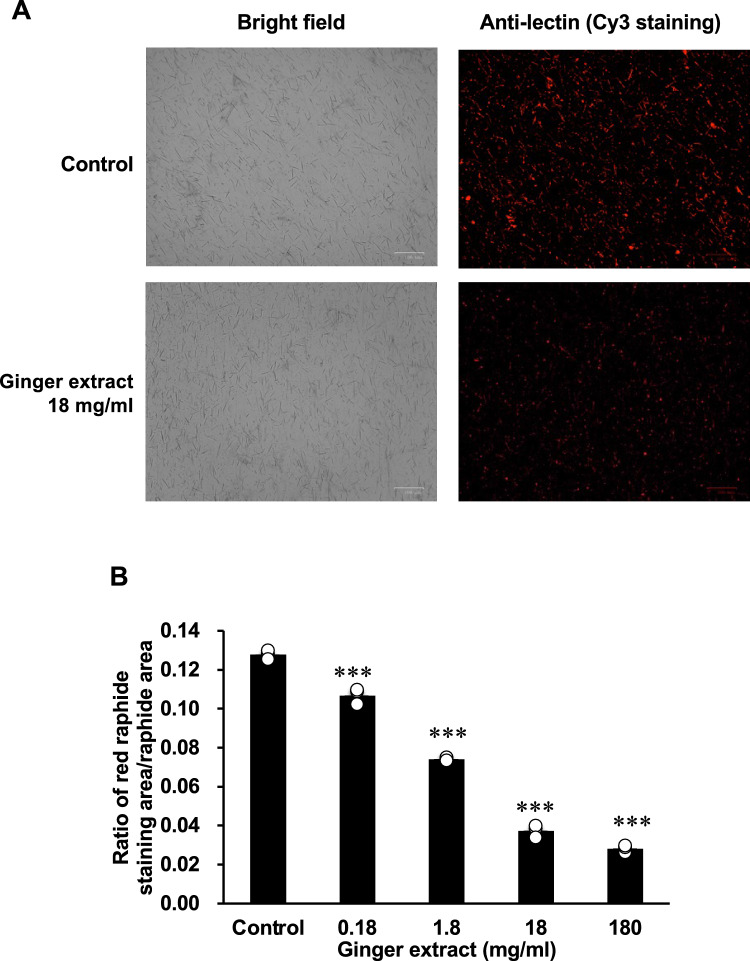
Immunostaining of PEX raphides of Pinellia tuber with the dried ginger extract using antiserum against *Pinellia ternata* lectin (PTL). PEX raphides were suspended in phosphate buffered saline (PBS) containing different concentrations of dried ginger extract and incubated at 40 °C for 30 min. Then, PEX raphides were stained with murine antiserum against PTL and Cy3-conjugated secondary antibody. **A** Photo of light and fluorescence microscopies. **B** The black area (raphides) in the photo of the light microscopies and the red area (PTL) in that of fluorescence microscopies were measured in the same position using Image J. The ratio of the red staining area to the raphide area was calculated. The results were presented as a bar graph showing the mean and individual values plotted ± S.D. (*n* = 3). ***p < 0.001 compared to the control group by Dunnet's multiple *t*-test. PEX, petroleum ether extraction

We treated PEX raphides with dried ginger extract and found that it reduced the amount of PTL at the surface of PEX raphides without affecting the shape and the number of raphides (Fig. 4A). By the dot blot analysis, we confirmed that dried ginger extract was not stained and that PTL was detected in the supernatants of the raphides treated with the dried ginger extract (Supplementary Fig. S2C and S2D). By the image analysis for the membrane, the amount of PTL in the supernatant of the raphide treated with dried ginger extract was significantly higher than that of the control (Supplementary Fig. S2E). The amount of PTL in the raphides was significantly reduced by the treatment of dried ginger extract in a concentration-dependent manner, and its IC_50_ value was 4.6 mg/ml (Fig. 4).

**Fig. 5:**
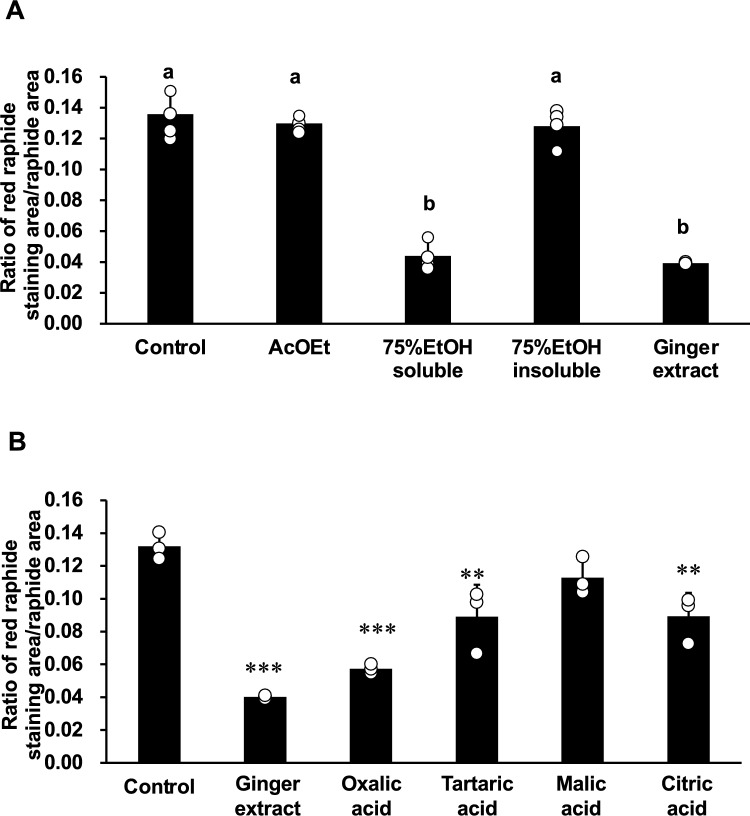
Effect of the fractions of dried ginger extract (**A**) or organic acids (**B**) on the immunostaining of PEX raphides of Pinellia tuber using antiserum against *Pinellia ternata* lectin (PTL). PEX raphides were suspended in phosphate buffered saline (PBS) containing the fractions of dried ginger extract or organic acids. The concentrations of each sample were adjusted to 18 mg/ml of ginger extract (oxalic acid (OX), 4.4 mM; tartaric acid (TA), 0.6 mM; malic acid (MA), 3 mM; citric acid (CA), 2.3 mM). The suspensions were incubated at 40 °C for 30 min. Then, the amount of PTL in PEX raphides was evaluated. The results were presented as a bar graph showing the mean and individual values plotted ± S.D. (*n* = 4 for A and 3 for B). **A** Different alphabetical letters a and b indicate statistically significant differences at *p* < 0.05 between each group evaluated by Bonferroni’s multiple comparison test. **B** **p < 0.01 and ***p < 0.001 compared to control group by Dunnet's multiple *t*-test. PEX, petroleum ether extraction

To predict the active ingredients contained in the dried ginger extract that reduced the amount of PTL in PEX raphides, we prepared the fractions of the extract, and treated PEX raphides with them at the concentration related to the original extract at 18 mg/ml. We found that the active ingredients were transferred into the 75% EtOH soluble part of the extract, and other fractions did not exhibit any effect on the PTL in PEX raphides (Fig. 5A).

We focused on organic acids as the active ingredients because the 75% EtOH soluble part would contain hydrophilic small molecular weight compounds, and our previous study revealed that the degenerating activity of dried ginger extract on PEX raphides was due to oxalic acid [13]. We measured the concentrations of organic acids in dried ginger extract and did not detect acetic acid, succinic acid, and maleic acid (their detection limits were less than 0.07%, 0.01%, and 0.09% (w/w), respectively, using ODS column in HPLC analysis. Tartaric acid, oxalic acid, and malic acid were eluted at 2.5–2.8 min and could not be quantified due to insufficient separation. Citric acid was detected at 4.3 min, and its content in dried ginger extract was 2.1% (w/w) (Supplementary Fig. S3A).

Then, we used HPLC analysis with a HILIC column to separate organic acids, and oxalic acid was detected at 8.6 min with good separation from other compounds. The content of oxalic acid in the dried ginger extract was 2.6% (w/w) (Supplementary Fig. S3B).

Tartaric acid and malic acid were analyzed using LC–MS with a specific column for organic acids. The contents of tartaric acid and malic acid in the dried ginger extract were 0.51% and 2.2% (w/w), respectively (Supplementary Fig. S3C).

**Fig. 6:**
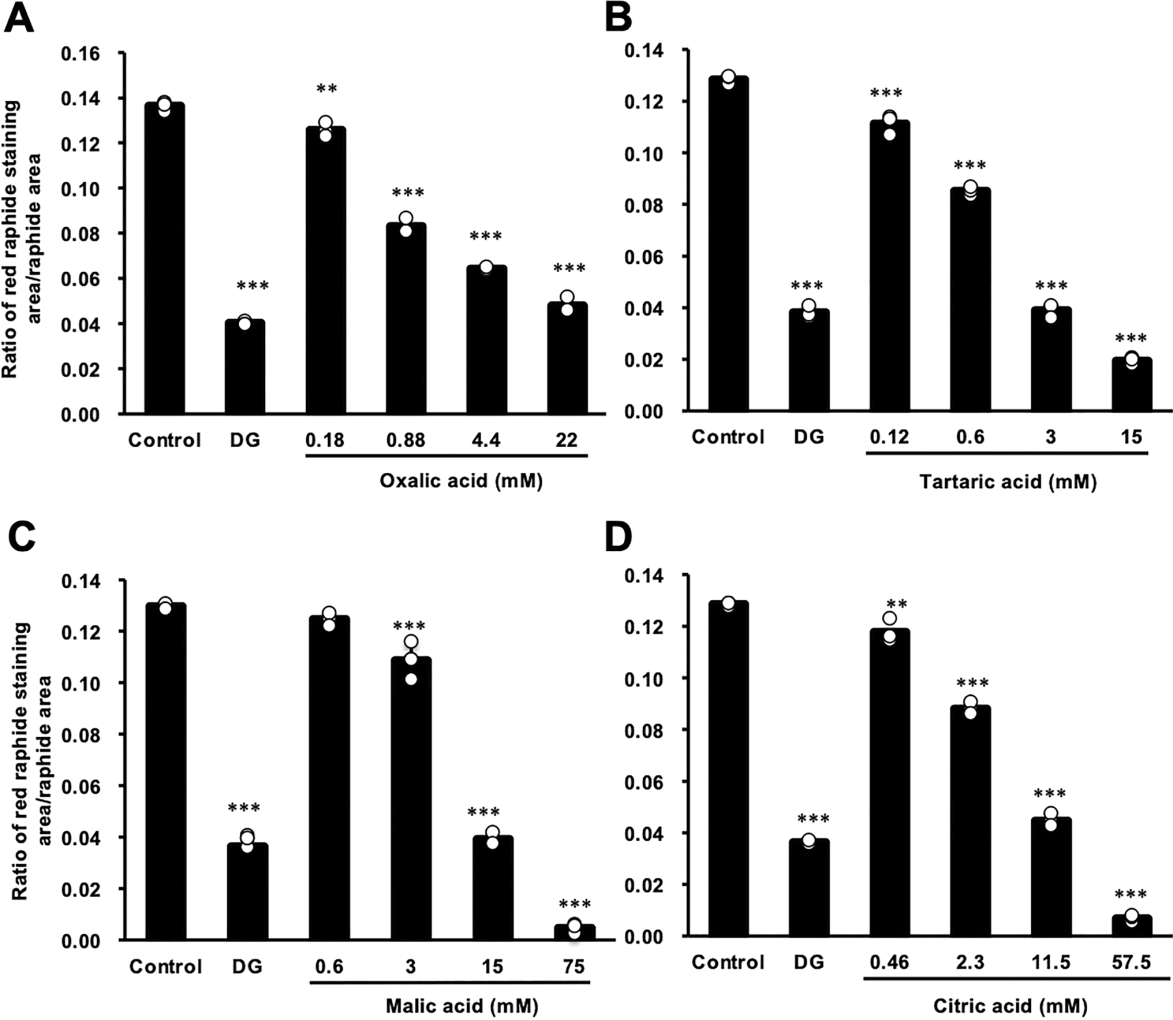
Effect of oxalic acid (**A**), tartaric acid (**B**), malic acid (**C**), and citric acid (**D**) on the immunostaining of PEX raphides of Pinellia tuber using antiserum against *Pinellia ternata* lectin (PTL). PEX raphides were suspended in phosphate buffered saline (PBS) containing various concentrations of organic acids. The concentration of dried ginger extract (DG) was 18 mg/ml. The suspensions were incubated at 40 °C for 30 min. Then, the amount of PTL in PEX raphides was evaluated. The results were presented as a bar graph showing the mean and individual values plotted ± S.D. (*n* = 3). **p < 0.01 and ***p < 0.001 compared to control group by Dunnet's multiple *t*-test. PEX, petroleum ether extraction

By preparing the solutions of four organic acids at concentrations equivalent to those in 18 mg/ml ginger extract, and treating with PEX, oxalic acid exhibited the best contribution to the reducing effect of PTL in PEX raphides, and tartaric acid and citric acid also exhibited significant effects (Fig. 5B). These organic acids exhibited a significant reducing effect on PTL in PEX raphides in concentration-dependent manners, and their IC_50_ values are 5.4 mg/ml of citric acid, 4.2 mg/ml of oxalic acid, 7.7 mg/ml of malic acid, and 1.3 mg/ml of tartaric acid, respectively (Fig. 6).

## Discussion

Recent studies have shown that the acridity of Pinellia tuber is caused by needle-like crystals, called raphides, composed of calcium oxalate and proteins [8, 14]. This acridity has been called as "toxicity," and there have been many descriptions of how to reduce this toxicity in successive textbooks in traditional Chinese medicine, one of which is processing with ginger juice [6]. However, in Japanese Kampo medicine, Shuan Kagawa (1683–1755) described that the "toxicity" of Pinellia tuber disappeared by decocting and Pinellia tuber should not be processed to avoid weakening its effectiveness [15]. Therefore, in the present Kampo medicine, Pinellia tuber is commonly used without processing for preparing decoctions [16].

It is reported that the origin of the toxicity of Pinellia tuber might be PTL. Lectins are defined as proteins possessing at least one non-catalytic domain, which bind reversibly to specific mono- or oligosaccharides [17]. Now more than 500 plant lectins have been studied in depth, leading to an updated definition of lectins in 2011 as a class of plant glycoproteins that have at least one non-catalytically active domain and reversibly bind to monosaccharides or oligosaccharides specifically [17]. In recent years, PTL has been reported as a novel plant-derived insecticidal lectin used to develop insect-resistant plants, and mannose-binding *P. ternata* leaf agglutinin has also been reported for its insecticidal activity [18]. Wu et al. found PTL in the raphides from Pinellia tuber by low-speed centrifugation of the suspension of Pinellia tuber and that PTL caused the inflammation by being dropped onto rabbit's eye [9]. However, they found other bands than PTL from the proteins of the raphides in their SDS-PAGE analysis since the purification method of the raphides using centrifugation must not be enough and may have been contaminated with other tissues and organs from the plant body. In our previous study, we found that the surface of the raphides of Pinellia tuber has a lipophilic character, and discovered the isolation method of raphides from powdered Pinellia tuber using PE extraction [10]. In the present study, we found that the raphides proteins exhibited the single band at 14 kDa containing two different protein sequences both of which were derived from PTL in SDS-PAGE analysis, and that the raphides can be immuno-stained by the antiserum against recombinant PTL. From these results, we can obtain the evidences that the raphides of Pinellia tuber contain PTL at their surface.

Regarding the mechanisms of "detoxication" for Pinellia tuber, it has been reported that the needle crystal can be destroyed through the solubilization of calcium oxalate by alum [19]. Indeed, alum can dissolve the raphides and we prepared the protein sample in PEX using aluminum ion according to the previous study of Yu et al. [20]. According to classical textbooks, ginger extract has been believed to counteract the toxicity of Pinellia tuber, and ginger extract and its component 6-gingerol exhibited a suppressive effect on the inflammation caused by Pinellia tuber [21]. In our previous study, we found that the lipophilic character of PEX raphides was denatured by heating for 30 min or by the treatment with dried ginger extract, and its acridity was reduced, although the shape of raphides observed by microscopy did not change [10]. The present study revealed that similar treatments reduced the content of PTL in the raphides, and the effect of dried ginger extract exhibited a concentration-dependent manner without heating. Furthermore, we confirmed that the heat-treatment and the treatment with dried ginger extract released PTL into the supernatant of the suspension from PEX raphides, respectively. It is suggested that the acridity of the raphides may be related to the content of PTL in the raphides, and that ginger may reduce the acridity of Pinellia tuber through a dual mechanism of anti-inflammatory action and reducing PTL proteins on the raphides. From the present results, it is predicted that PTL must be contained in the decoctions of Pinellia tuber, but we did not find any throat acridity in the decoctions [15]. Heat treatment not only makes PTL released from the raphides but may denature PTL not to cause throat acridity.

We tried to find the active ingredients in a dried ginger extract that could reduce the content of PTL in the raphides of Pinellia tuber. Through activity-guided fractionation, we found that the activity was transferred to the 75% ethanol-soluble fraction. This fraction would contain hydrophilic low molecular weight compounds, such as amino acids, monosaccharides, disaccharides, anions, cations, and others. Since we had determined that the active component in a dried ginger extract that denatured the PEX raphides was mainly oxalic acid in our previous study [13]. Therefore, we focused on organic acids as potential candidates that contribute to the activity of the dried ginger extract. We analyzed the concentrations of organic acids in the dried ginger extract, and measured the activities of each organic acid at concentrations related to the dried ginger extract at 18 mg/ml. Then, oxalic acid, tartaric acid, and citric acid exhibited a significant effect on the reduction of PTL in the raphides, though malic acid did not. Since the sum of the activities of oxalic acid, tartaric acid, and citric acid was higher than that of dried ginger extract, most of the activity of dried ginger extract could be explained by these three organic acids. Although tartaric acid exhibited the strongest activity among the components in dried ginger extract, the concentration of tartaric acid in the extract was lower than that of oxalic acid, and the contribution of oxalic acid was the greatest among these organic acids. This result is consistent with our previous research about the denaturing activity of the hydrophobicity of the raphides [13]. In our previous study using "Fabanxia", the processed Pinellia tuber with licorice decoction, glycyrrhizin and glycyrrhetinic acid exhibited the denaturing activity of the hydrophobicity of the raphides but glucuronic acid did not at the concentration of 1 mM [22]. These results suggest that not all organic acids but those with specific chemical structure would exhibit the denaturing activity of the raphides. Since the recombinant PTL is hard to dissolve in H_2_O but can dissolve in PBS and PE (data not shown), the hydrophobicity of the surface of the raphides derived from PTL, the denaturing activity of organic acids on the raphides, and the release of PTL from the raphides must be related closely. In our previous study, we confirmed that α-starch contributed to the denaturing of the hydrophobicity of the raphides [22]. Therefore, when PEX raphides are existing in Pinellia tuber in the processing using ginger, starch may contribute to the denaturing of the raphides in addition to organic acids in ginger.

PTL isolated in the present study contained 810 bp open reading frame encoding 269 amino acids, and was 94.4% identical to the original amino acid sequence of EU199445.1. PTL has high homology with other mannose-binding plant lectin genes, with three mannose-binding sites, and the sequences of these sites are highly conserved with the first two binding sites being QDNY and the third being QDFY [12, 23]. Although a mutation was observed in PTL isolated, these sites are still conserved, and the function of mannose-binding lectins in monocotyledons would be observed. The mature PTL expressed in *E. coli* was approximately 28 kDa. Therefore, the PTL in the raphides may bind to the raphides after cleaved in the *Pinellia* cells because the size of the PTL obtained from the raphides was approximately 14 kDa.

It is said that the acridity of the needle-shaped crystal in the plant belonging to Araceae family is caused by the puncture of the needle-shaped crystal through the cell membrane of the throat epithelial cells [24]. However, the puncture of the needle through the cell membrane must not be the cause of the acridity because the raphides heated or treated with dried ginger extract have the same needle-shaped crystal but do not cause throat acridity according to our previous studies [10, 13]. Actually, there is no evidences to support the hypothesis that needle-shaped crystals penetrate the cell membrane. If the needle-shaped crystals penetrate the cell membrane to cause the acridity, the needle-shape may play an important role as a transporter of PTL into the inside of the epithelial cells. And we are considering another possibility of the mechanism to cause the throat acridity that the raphides adhere to the cell surface and PTL may leak out from the raphides to activate some receptors or channels on the cell membrane. Indeed, it is reported that PTL can cause inflammation by stimulating toll-like receptor 4 [25]. We will verify this in future research.

In conclusion, we prepared the antiserum against recombinant PTL and used it to verify that the raphides in Pinellia tuber contain PTL by immunostaining PTL in the raphides is reduced by heating or processing with dried ginger extract, and these phenomena might be related to reduce the throat acridity of the raphides of Pinellia tuber. Furthermore, we found oxalic acid, tartaric acid, and citric acid as the active ingredients of dried ginger extract to reduce PTL in the raphides of Pinellia tuber. The present study exhibits scientific evidences supporting the traditional theories of processing to "detoxify" Pinellia tuber.

### Supplementary Information

Below is the link to the electronic supplementary material.

Supplementary file1 (PDF 52 KB)

Supplementary file2 (PDF 1038 KB)

## Data Availability

The data used to support the findings of this study are available from the corresponding author upon request.
