# Correction: Heating or ginger extract reduces the content of *Pinellia ternata* lectin in the raphides of Pinellia tuber

**DOI:** 10.1007/s11418-024-01808-z

**Published:** 2024-04-03

**Authors:** Yan Liu, Itsuki Nose, Kazuyoshi Terasaka, Tsukasa Fueki, Toshiaki Makino

**Affiliations:** 1 Department of Pharmacognosy, Graduate School of Pharmaceutical Sciences, Nagoya City University, 3-1 Tanabe-dori, Mizuho-ku, Nagoya, 467-8603 Japan; 2 Matsuya Pharmacy, 2927-5 Maki-kou, Nishikan-ku, Niigata, 953-0041 Japan; 3 Department of Traditional Medicine, Toho University School of Medicine, 5-21-16, Omori Nishi, Ota-ku, Tokyo, 143-8540 Japan

**Correction to: Journal of Natural Medicines (2023) 77:761–773** 10.1007/s11418-023-01717-7

The article Heating or ginger extract reduces the content of *Pinellia ternata lectin* in the raphides of Pinellia tuber, written by Yan Liu, Itsuki Nose, Kazuyoshi Terasaka, Tsukasa Fueki, Toshiaki Makino, was originally published Online First without Open Access. After publication in volume 77, issue 4, page 761–773 the author decided to opt for Open Choice and to make the article an Open Access publication. Therefore, the copyright of the article has been changed to © The Author(s) 2024 and the article is forthwith distributed under the terms of the Creative Commons Attribution 4.0 International License, which permits use, sharing, adaptation, distribution and reproduction in any medium or format, as long as you give appropriate credit to the original author(s) and the source, provide a link to the Creative Commons license, and indicate if changes were made. The images or other third party material in this article are included in the article’s Creative Commons licence, unless indicated otherwise in a credit line to the material. If material is not included in the article’s Creative Commons licence and your intended use is not permitted by statutory regulation or exceeds the permitted use, you will need to obtain permission directly from the copyright holder. To view a copy of this licence, visit http://creativecommons.org/licenses/by/4.0.

The original article was updated.

