# Supplementary material for: Heating or ginger extract reduces the content of Pinellia ternata lectin in the raphides of Pinellia tuber
Source: J Nat Med. 2023 Jun 13;77(4):761–73. doi: 10.1007/s11418-023-01717-7 (PMC10987350; doi:10.1007/s11418-023-01717-7)
Supplement: Supplementary file 1 — Supplementary file1 (PDF 52 KB) [file 11418_2023_1717_MOESM1_ESM.pdf]

Supplementary Table S1. Primers used in cDNA cloning and vector construction of *Pinellia ternata* lectin (PTL)

Primer #1 (sense)	ATGGCCTCCAAGCTCCTCCTCTTC
Primer #2 (antisense)	TTAATTCACCTTCTCCGTCACCATGCC
Primer #3 (sense+ BamHI)	ACAAGAGTCCGGATCCC
Primer #4 (antisense+XhoI)	CTTTACCAGACTCGAGTTA

Primers #1 and #2 were for the open reading frame sequence designed based on PTL mRNA.
Primers #3 and #4 were for the subcloning PTL into pET45b.
