# Supplementary material for: Heating or ginger extract reduces the content of Pinellia ternata lectin in the raphides of Pinellia tuber
Source: J Nat Med. 2023 Jun 13;77(4):761–73. doi: 10.1007/s11418-023-01717-7 (PMC10987350; doi:10.1007/s11418-023-01717-7)
Supplement: Supplementary file 2 — Supplementary file2 (PDF 1038 KB) [file 11418_2023_1717_MOESM2_ESM.pdf]

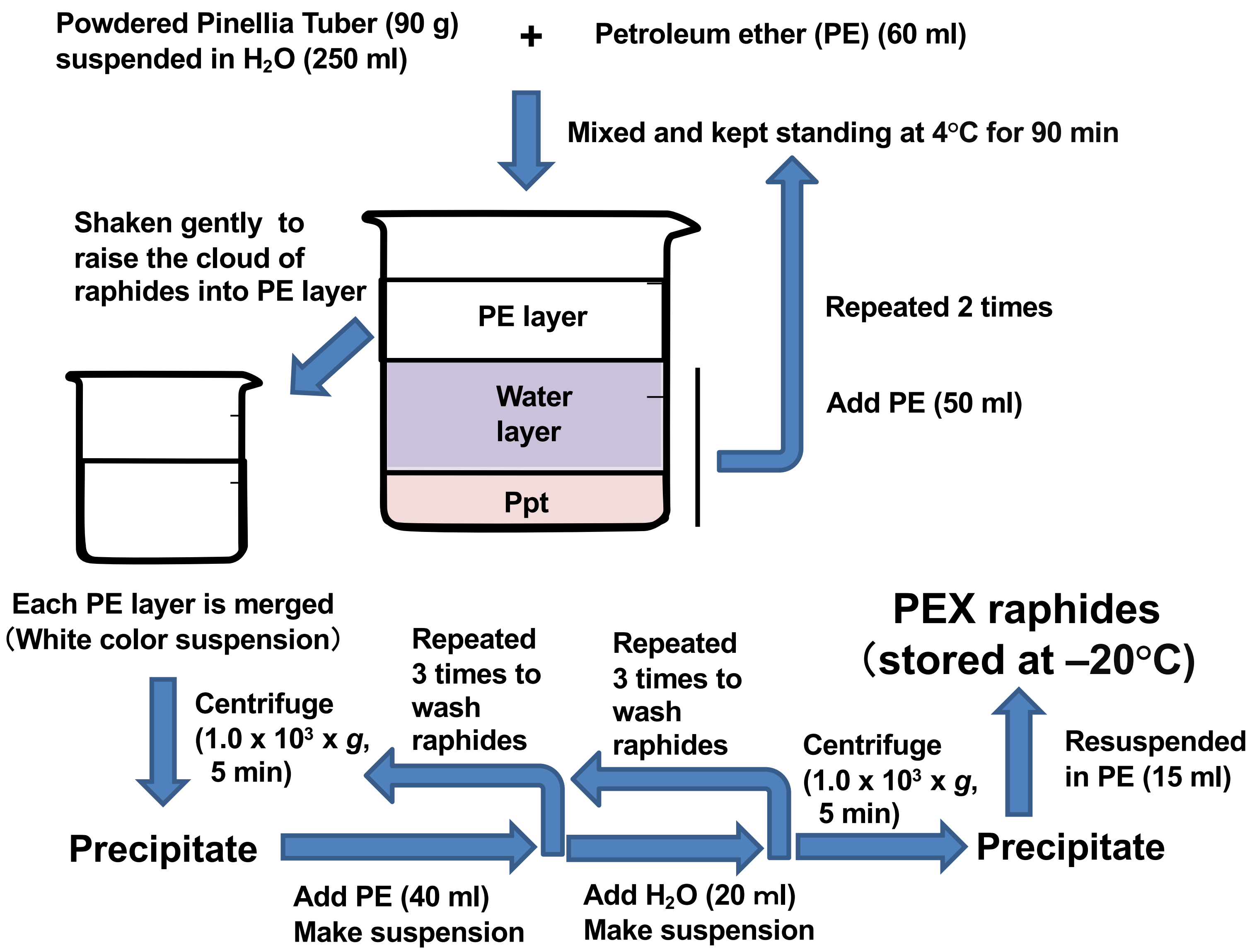

Supplementary Fig. S1. The scheme of the preparation for PE extract (PEX) raphides from powdered Pinellia Tuber.

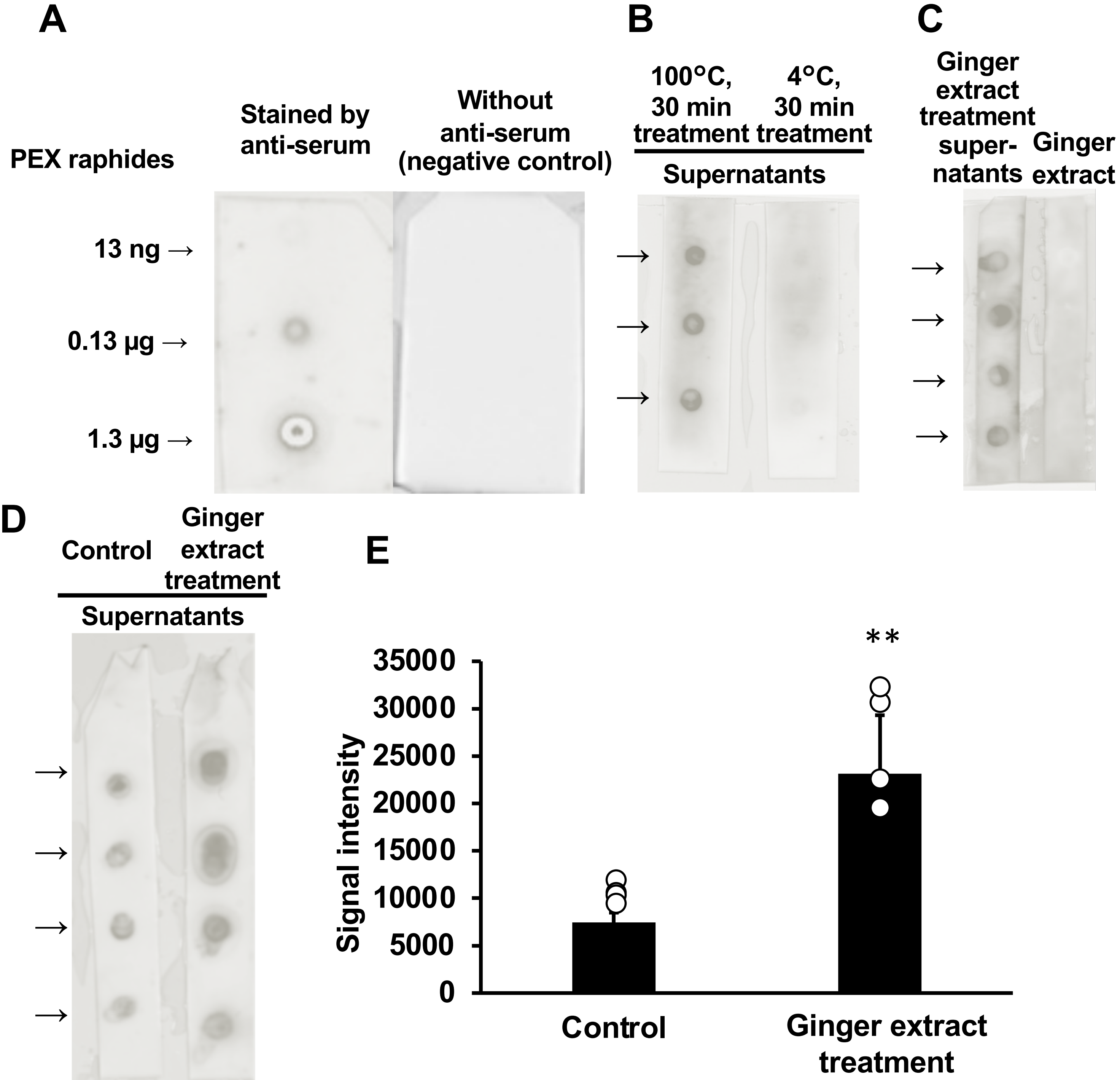

Supplementary Fig. S2. Dot blot analysis of PEX raphides and the supernatant of PEX raphides treated with heat or ginger extract. (A) The homogenate of PEX raphides was spotted onto the membrane. Then, the membrane was stained with anti-serum against recombinant *Pinellia ternata* lectin. The right membrane is the negative control stained with second antibody without anti-serum. (B) The supernatant (5 µl) of the suspension of PEX raphides treated with 100°C or 4°C was spotted onto the membrane. (C) The supernatant (5 µl) of the suspension of PEX raphides treated with the dried ginger extract (18 mg/ml) was spotted (left). The dried ginger extract (18 mg/ml, 5 µl) was spotted (right). (D) The supernatant (5 µl) of the suspension of PEX raphides treated with PBS (for control, left) or the dried ginger extract (18 mg/ml, right, the same sample of C light membrane) was spotted. (E) The area of the spot stained in the membrane D were measured using Image J, and the results were presented as a bar graph showing the mean and individual values plotted ± standard deviation (S.D.) ($n = 4$). **$p < 0.01$ compared to control group by Student's *t*-test. Since the staining areas in the right membranes in A, B, and C were undetectable by Image J, respectively, the quantitative analysis could not performed in A, B, and C.

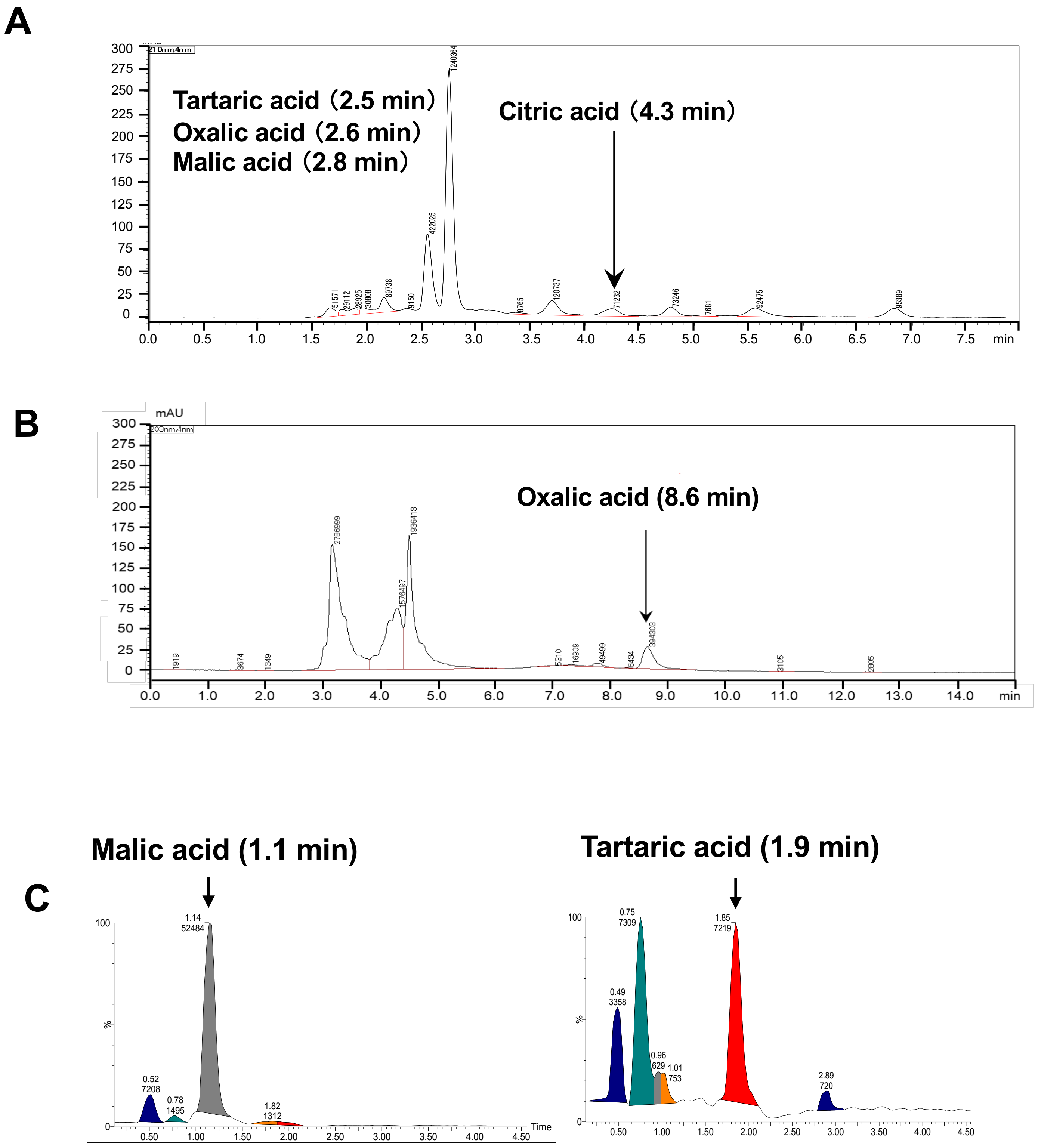

Supplementary Fig. S3. HPLC chromatograms of organic acids in ginger extract. (A) Column, Cosmosil 5C18-PAQ (150 mm × 4.6 mm i.d., Nacalai Tesque); mobile phase, phosphate buffer (10 mM, pH 2.5), 1.0 ml/min; detection, UV 210 nm; column temperature, 30°C. (B) Column, Cosmosil HILIC (250 mm × 4.6 mm i.d., Nacalai); mobile phase, 10 mM phosphate buffer (pH 6.8)/acetonitrile (70:30), 1 ml/min; detection, UV 203 nm; column temperature, 40°C. (C) Column, Intrada Organic Acid (50 × 2 mm i.d., Intakt); mobile phase, acetonitrile/H_2O/formic acid = 10/90/0.1 (A) and acetonitrile/100 mM ammonium formate = 10/90 (B), 0% B (0 – 1 min), 0 – 100% B (1 – 7 min), 0.2 ml/min; column temperature, 25°C; detection, ESI(–)SIM analysis, malic acid (*m/z* = 133.0) and tartaric acid (*m/z* = 149.0).
